# A Tale about *Shigella*: Evolution, Plasmid, and Virulence

**DOI:** 10.3390/microorganisms11071709

**Published:** 2023-06-30

**Authors:** Nathaline Haidar-Ahmad, France Ourida Manigat, Navoun Silué, Stéphanie M. Pontier, François-Xavier Campbell-Valois

**Affiliations:** 1 Host-Microbe Interactions Laboratory, Centre for Chemical and Synthetic Biology, Department of Chemistry and Biomolecular Sciences, University of Ottawa, Ottawa, ON K1N 6N5, Canada; nhaid010@uottawa.ca (N.H.-A.); fmani073@uottawa.ca (F.O.M.); nsilu022@uottawa.ca (N.S.); 2 Centre de Recherche Santé Environnementale et Biodiversité de l’Outaouais (SEBO), CEGEP de l’Outaouais, Gatineau, QC J8Y 6M4, Canada; stephanie.pontier@cegepoutaouais.qc.ca; 3 Centre for Infection, Immunity and Inflammation, Department of Biochemistry, Microbiology and Immunology, University of Ottawa, Ottawa, ON K1N 6N5, Canada

**Keywords:** *Shigella*, *Escherichia coli*, pathogenesis, plasmid, type III secretion system, cell-to-cell spread, regulation of virulence genes, VirF, VirB, MxiE

## Abstract

*Shigella* spp. cause hundreds of millions of intestinal infections each year. They target the mucosa of the human colon and are an important model of intracellular bacterial pathogenesis. *Shigella* is a pathovar of *Escherichia coli* that is characterized by the presence of a large invasion plasmid, pINV, which encodes the characteristic type III secretion system and *icsA* used for cytosol invasion and cell-to-cell spread, respectively. First, we review recent advances in the genetic aspects of *Shigella*, shedding light on its evolutionary history within the *E. coli* lineage and its relationship to the acquisition of pINV. We then discuss recent insights into the processes that allow for the maintenance of pINV. Finally, we describe the role of the transcription activators VirF, VirB, and MxiE in the major virulence gene regulatory cascades that control the expression of the type III secretion system and *icsA*. This provides an opportunity to examine the interplay between these pINV-encoded transcriptional activators and numerous chromosome-encoded factors that modulate their activity. Finally, we discuss novel chromosomal genes *icaR*, *icaT*, and *yccE* that are regulated by MxiE. This review emphasizes the notion that *Shigella* and *E. coli* have walked the fine line between commensalism and pathogenesis for much of their history.

## 1. Introduction

Many gram-negative bacteria, such as *Bordetella*, *Burkholderia*, *Citrobacter*, *Chlamydia*, *Escherichia*, *Pseudomonas*, *Rhizobium*, *Salmonella*, *Shigella*, *Xanthomonas*, and *Yersinia*, interact with host cells using the syringe-shaped type III secretion system (T3SS) also known as the injectisome. This large proteinaceous complex injects substrate proteins into host cells to hijack them for the benefit of the bacteria that harbor it. Since 2015, spectacular progress has been made in describing its structure and function [1,2,3,4,5,6,7,8,9]. The T3SS is composed of the sorting platform that selects the protein substrates in the bacterial cytosol, the transmembrane needle complex that serves as a channel for the secretion of the substrates, the tip complex that triggers secretion upon sensing the physical contact with host cells, and the translocon that establishes the continuity between the needle complex and the host cytosol during the delivery of the substrates [7]. The *Shigella flexneri* T3SS has been one of the most extensively studied, providing us with an in-depth understanding of its role and function in pathogenesis (reviewed in [10,11,12]). In *Shigella*, the fate of the T3SS is tied to the large invasion plasmid that encodes it. Acquisition of this plasmid drove the evolution of *Shigella* spp. from *Escherichia coli*. This makes *Shigella* a powerful model to study the evolution of virulence and the interplay of the T3SS with its genome. Here, we focus on the emergence of the *Shigella* pathovar, the maintenance of the invasion plasmid, and the gene regulatory mechanisms that control the expression of the T3SS and of the cell-to-cell spread traits.

## 2. The Evolution of the *Shigella* Pathovar

*Shigella* spp. are gram-negative enterobacteria divided in four subgroups named *S. boydii*, *S. dysenteriae*, *S. flexneri*, and *S. sonnei*. Their unique pathogenesis, their lack of flagellar motility and inability to ferment lactose constitutes the hallmark of the *Shigella* genus established in the 1950s [13]. However, modern phylogeny approaches revealed that *Shigella* spp. are merely an *Escherichia coli* pathovar [14]. *Shigella* spp. infect the large intestine of humans and cause diarrheal symptoms ranging from watery to mucopurulent and bloody stools accompanied by inflammation (also known as dysentery). *Shigella* spp. cause 80–190 million infections [15,16], and approximately 200,000 deaths annually, with more than 50% of cases occurring in individuals younger than 5 years or older than 70 years [17]. *S. flexneri* is responsible for most cases and, similar to *S. dysenteriae*, is more common in low-income countries. By contrast, *S. sonnei* is responsible for most cases in high-income countries. [18]. Colony-forming units in the low hundreds can cause the disease in healthy humans [19], making *Shigella* more infectious than most other enterobacteria. *Shigella* has no known animal reservoirs and is therefore considered to be human specific. It is transmitted from person to person or by ingestion of contaminated water or food. As with other enterobacteria [20,21,22,23,24], increasing antibiotic resistance in *Shigella* spp. is a public health concern [20,25,26,27].

From an evolutionary perspective, *Shigella* emerged 35,000–270,000 years ago [28,29], contemporaneously with *Homo sapiens*. The evolutionary trajectories of *S. dysenteriae*, *S. flexneri*, and *S. sonnei*. are unique [30,31,32]. In the case of *S. dysenteriae* and *S. sonnei*, modern historical events and societal changes have greatly accelerated their expansion and spread. *Shigella* spp. have adapted to their human host by losing or inactivating chromosomal genes detrimental to pathogenesis [33,34]. Thus, the number of chromosomal genes in *Shigella* is reduced compared to their commensalistic *E. coli* counterparts, a phenomenon shared with several bacterial pathogens. In a remarkable example of convergent evolution, some *Shigella* strains lost the same chromosomal genes through disruption by discrete insertion sequences [28,35]. Thus, an intriguing question about *Shigella* is whether it emerged once [36], or on multiple occasions [28] within the *E.* coli lineage. Since these seminal studies, the number of sequenced *E. coli* genomes has increased dramatically, allowing further study of their phylogeny [37,38,39]. For example, analysis of 10,667 chromosome sequences, including 1283 from *Shigella* spp., using the fast distance estimation method MASH revealed 14 *E. coli* phylogroups [37]. Two of these, named Shig1 and Shig2, consisted exclusively of *Shigella* strains, with an overrepresentation of *S. flexneri* and *S. sonnei*, respectively, and contained most of the *Shigella* genomes in the dataset (Table 1). This analysis confirmed on an unprecedented scale that *Shigella* shared several of the hallmarks of bacterial pathogens. Indeed, Shig1 and Shig2 strains had a lower guanine-cytosine content, smaller genomes, and higher rates of gene loss and duplication on average than other *E. coli* phylogroups. The analyses also revealed that *S. sonnei* in Shig2 possesses a unique set of core genes that are not conserved in other phylogroups, highlighting its clonality and unique evolutionary origin. Nevertheless, *Shigella strains* were also found in seven other phylogroups (Table 1), with B1 being the most dominant. Interestingly, analyses measuring the conservation and loss of protein families confirmed the remarkable convergent evolution of Shig1 and *Shigella* from the B1 phylogroup. Taken together, this study provides a comprehensive overview of the diversity of *Shigella*, supporting the notion that it emerged multiple times.

**Figure 1:**
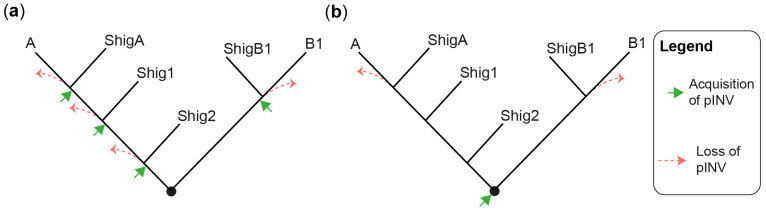
Models for the emergence of *Shigella* from *E. coli*. (**a**) A tree illustrating the multiple pINV acquisition model. (**b**) A tree illustrating the single pINV acquisition model. These phylogenetic trees are qualitative and intended to be used for the sole purpose of illustrating the main difference between the two models in a straightforward manner. ShigA: *Shigella* from phylogroup A; ShigB1: *Shigella* from phylogroup B1; Shig1 and Shig2 as defined in the text and Table 1.

The evolution and pathogenesis of *Shigella* spp. and of the less virulent enteroinvasive *E. coli* (EIEC) is associated with the acquisition of a large invasion plasmid of 220 kbp, called pINV, which has a high degree of sequence conservation across strains [30,40,41,42,43]. The genome remodeling in EIECs is less pronounced than in *Shigella*. Therefore, EIECs can be described as an intermediate between commensal *E. coli* and *Shigella*, which may help us to understand the evolution of the *Shigella*-EIEC pathotype and the coevolution of the chromosome with pINV [42]. Since pINV played a key role in the emergence of *Shigella* and EIEC clades, each of them should have originated from an independent pINV acquisition event (Figure 1a). Although a consensus has emerged around this model [28,29,37,44], the fact that pINV is not conjugative, and therefore not susceptible to horizontal transfers, is a hurdle that, to our knowledge, has not yet been addressed. The alternative model proposes that pINV was acquired once by a common ancestor of all *Shigella* and a subset of *E. coli* [36] (Figure 1b). *Shigella* would have conserved pINV by adapting their genome to the pathogenic lifestyle, whereas non-EIEC *E. coli* would have lost it by failing to make these adaptations. This model accounts for the non-transmissibility of pINV, but it has its own set of challenges. Not least of these is that it requires the relatively unstable pINV to have been maintained over the evolutionary time required for the emergence of *Shigella* spp. Despite these limitations, we believe that the single acquisition model, or a hybrid model in which related *Shigella* clades may be the result of a single pINV acquisition event, should be considered in light of the large number of new sequences obtained since it was first described [36].

## 3. The Invasion Plasmid pINV

The discovery of pINV in the 1980s marked the beginning of the molecular era in the study of shigellosis [13]. The key feature of pINV is the pathogenicity island, called the entry region, which contains the *mxi*, *spa*, and *ipa* operons that encode the parts of the injectisome and the translocators needed for host cell invasion. Once *Shigella* reaches the host cytosol, the outer membrane protein IcsA, also encoded by pINV, propels *Shigella* through the formation of actin comets, which are critical for invading neighboring cells [45]. Other bacteria possessing a T3SS have operons similar to those of the entry region, although their genetic organization and names differ [7,46,47]. IcsA is unique to pINV, although unrelated proteins confer similar actin-mediated cell-to-cell spreading abilities to other bacteria [48]. Together, the entry region and *icsA* are required for the hallmark invasiveness of *Shigella*. In addition, several effector genes are distributed throughout the rest of the pINV, which abounds in insertion sequences that represent approximately 25% of the total sequence [40]. pINV also harbors genes unrelated to the T3SS that are transcribed in regular growth medium [41,49], but their contribution to pathogenesis is poorly characterized with few exceptions [50,51,52,53]. The pINV exists in two forms, designated A and B [54], and its phylogeny suggests that it was obtained through horizontal transfers. Since pINV is not conjugative, the mechanism of these transfers is unclear. However, one study reported that transfers of non-conjugative plasmids could occur at a low rate in *E. coli* biofilms [55]. To our knowledge, this phenomenon has not been implicated in the transfer of pINV.

**Figure 2:**
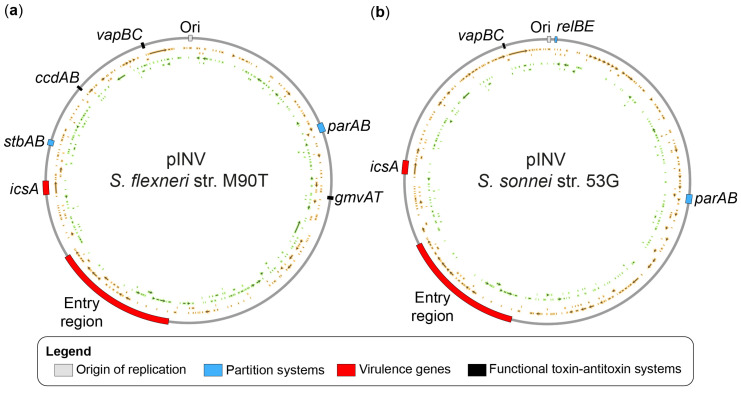
The invasion plasmid pINV. (**a**) pINV from *S. flexneri* strain M90T (accession number NC_024996.1). (**b**) pINV from *S. sonnei* strain 53 G (accession number NC_016833). The main features indicated in the outer rim are described in the legend. The open reading frames are represented by arrows and rectangles in the inner rims: the rims colored orange represent the top DNA strand, and the rims colored green represent the bottom DNA strand. The plasmid maps were created with Snapgene and Illustrator.

The pINV is present in one to two copies per cell. Expression of T3SS-related genes renders the pINV unstable [56], resulting in a high rate of total or partial loss in the population. Nevertheless, *S. flexneri* uses five systems to maintain pINV. These include two partitioning systems *parAB* and *stbAB*, and three type II toxin–antitoxin (TA) systems *vapBC* (also known as *mvpAT*), *ccdAB*, and *gmvAT* [57]. By contrast, *S. sonnei* lacks *ccdAB*, *gmvAT*, and *stbAB*, and instead harbors the *relBE* partitioning system [58] (Figure 2). In *S. flexneri*, *vapBC* plays a dominant role in the stabilization of pINV at 37 °C, whereas *gmvAT* is the most important at 21 °C [59,60]. Although *ccdAB* is fully functional, its role can be masked by the presence of *gmvAT* and *vapBC*, suggesting that the latter two play a dominant role in the in vitro conditions tested. In the absence of the TA systems, ParAB stabilized the plasmid at 37 °C, whereas StbAB stabilized it at 21 °C in Δ*parAB*. These observations highlight the partial redundancy of these systems [60]. Both the ParAB and StbAB partitioning systems are functional, although their contribution to the stabilization of pINV in the presence of the TA systems is also undetectable [59]. The ability of type II TA systems to maintain a plasmid is based on the intrinsic toxicity of its toxin moiety and the shorter half-life of its antitoxin moiety [61,62]. When a daughter cell acquires a plasmid containing a type II TA system, the toxin is sequestered by the continued expression of the antitoxin. On the other hand, the loss of the plasmid reduces the amount of antitoxin, thereby killing the unfortunate daughter cell through the unchecked activity of the released toxin. This suggests that a protease must control the half-life of antitoxins. Genetic dissections suggested that both *vapBC* and *gmvAT* are regulated by *lon* [59]. Accordingly, a *S. flexneri* strain lacking the lon protease lost pINV 100 times more frequently.

In addition, the position of the locus of the TA systems was found to influence the nature of pINV remodeling that leads to the loss of virulence [63]. In *S. flexneri*, the loss of virulence was mostly mediated by the deletion of the entry region through recombination events involving flanking insertion sequences. The displacement of *vapBC* near the entry region abolished most of these recombination events [63]. Thus, in addition to its global effect on the stability of pINV, *vapBC* can exert a local stabilizing effect on neighboring genes. This means that the variation in the position of the TA systems could have consequences, although its relevance in *Shigella* is unknown. Indeed, it is noteworthy that the position of *vapBC* is conserved in *S. flexneri* and *S. sonnei* (Figure 2). Finally, this study also characterized the reversible integration on pINV in the chromosome through the recombination of insertion sequences. This suggests a pathway for the transfer of pathogenicity islands from the plasmid to the chromosome. 

The absence of *gmvAT* in *S. sonnei* destabilizes its pINV at room temperature [60]. Thus, the instability of pINV when *S. sonnei* is not in its host may explain why it is often transmitted from person to person [60]. It is arguable that the specific characteristics of *S. sonnei*’s pINV may allow further evolution of this lineage into an obligate pathogenic lifestyle. Interestingly, the reintroduction of the two missing TA systems *ccdAB* and *gmvAT* in *S. sonnei* stabilized its pINV, but not to the same extent as in *S. flexneri* [58]. A role for the *relBE* partitioning system unique to *S. sonnei* was rejected under the conditions tested. Instead, the data suggest that a K32R polymorphism in the toxin VapC toxin contributes to the instability of *S. sonnei*’s pINV. How this mutation affects the activity of VapBC is unknown, but a reduction in the endonuclease activity of the toxin was ruled out [58]. 

Taken together, the diverse combination of maintenance systems of the *sonnei* and the *flexneri* subgroups determines the stability of their respective versions of pINV, which affects their pathogenesis and epidemiology. Accordingly, these maintenance systems are practical for typing *Shigella* strains because they are better conserved than T3SS-related genes and reproduces the phylogeny obtained using chromosomal genes [64]. This supports the importance of these maintenance systems in the pathogenesis and the evolution of *Shigella*.

## 4. The Major Virulence Gene Regulatory Cascade in *Shigella*

**Figure 3:**
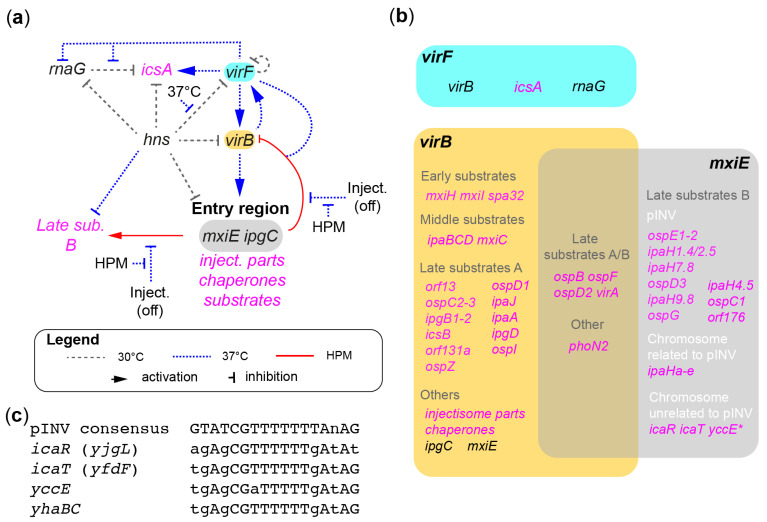
The major gene regulatory cascade in *Shigella*. (**a**) The signaling network regulating the expression of the invasive (T3SS) and cell-to-cell spread (*icsA*) traits in *Shigella*. It is centered around a vertical axis composed of transcription activators *virF*, *virB*, and *mxiE* and negatively regulated by *hns* at 30 °C. Events dominating at 30 °C, 37 °C, and when the injectisomes (inject.) are activated by contact with the HPM are represented as indicated in the legend. The transcription regulators and their terminal targets are written in black and magenta, respectively. (**b**) The *virF*, *virB*, and *mxiE* regulon. (**c**) The MxiE boxes of novel chromosomal genes *icaR*, *icaT*, *yccE*, and *yhaBC* compared with the consensus MxiE box of pINV-related members of the MxiE regulon.

The recent acquisition of pINV provides an opportunity to inspect its interaction with the chromosome at the gene expression level. In this section, we will focus on factors that directly control the expression of T3SS-related genes and *icsA*. Their expression is regulated by the temperature and the activation of the injectisomes. This occurs mainly at the transcriptional level through a virulence gene regulatory cascade consisting of the histone-like nucleoid structuring protein (H-NS) encoded in the chromosome, and the transcriptional activators VirF, VirB, and MxiE encoded in pINV (Figure 3a,b). 

H-NS is the negative regulator of the cascade. It is a DNA-binding protein that silences the expression of horizontally transferred genes, which is also known as xenogeneic silencing [65]. For example, *E. coli* lacking H-NS increased expression of xenogenes with a high adenine-thymine content [66]. The structure of H-NS associated with DNA is unknown, but molecular dynamics predicted that H-NS uses a three-residue motif to bind the minor groove of DNA in adenine-thymine rich stretches separated by 8–17 base pairs in two possible binding modes [67,68]. Indeed, H-NS can bind a single stretch of DNA through a stiffening linear mode, which is dominant at physiological magnesium concentrations, or two stretches of DNA through a bridging mode, which is stabilized at supraphysiological magnesium concentrations [69]. H-NS can repress transcription initiation regardless of the binding mode used, while the bridging mode is hypothesized to also inhibit transcription elongation [68]. H-NS has multiple validated and potential binding sites in the entry region as well as in other regions of pINV and in T3SS-related chromosomal genes with a high adenine-thymine content (~65%). Indeed, H-NS silences the promoters of *virF*, *virB*, and most T3SS-related genes at room temperature (<32 °C) [65,70,71,72,73]. At temperatures similar to those of its host (37 °C), the binding of H-NS to the *virF* promoter is weakened, thereby allowing the production of the VirF protein [72,73]. A shorter version of VirF (VirF_21_; MW 21 kDa), generated from an alternative translation initiation codon also contributes to repressing the production of the full-length VirF protein at 30 °C [74,75]. This regulatory mechanism may contribute to maintaining tight control of virulence, but its role in pathogenesis is unknown.

The first step in the gene regulatory cascade is controlled by VirF. It is a transcriptional activator of the AraC family that stimulates the expression of *virB* and *icsA* at 37 °C [73] (Figure 3a,b). A minor role of VirF in the regulation of *icsP*, which encodes a post-translational regulator of *icsA*, has also been reported [76]. Thus, the regulation of these genes by VirF allows for the coordination of the expression of the invasion and cell-to-cell spreading traits, which, if you recall, are hallmarks of *Shigella* pathogenesis. VirF and H-NS have several partially overlapping binding sites around or downstream of the −10 box of *icsA* and its small RNA attenuator RnaG [77]. RnaG attenuates *icsA* expression at 30 °C and to some extent at 37 °C as well. In contrast, H-NS silences *icsA* at 30 °C, whereas at 37 °C VirF relieves H-NS-mediated silencing, thereby upregulating *icsA* transcription [77]. At 37 °C, VirF may also repress the expression of *rnaG* [77] and stabilize *icsA* mRNA by disrupting the transcription attenuation duplex the latter can form with RnaG [78]. Taken together, these studies suggest that VirF dual-activates *icsA* through the release of both H-NS transcriptional repression and RnaG attenuation of its mRNA. Similarly, *virB* is positively regulated by VirF and negatively regulated by H-NS [70]. However, the mechanism by which VirF activates *virB* has unique features. First, there is evidence that VirF must form a dimer to bind *icsA*, whereas this is not required for its binding to *virB* or *rnaG* [79,80]. Second, two DNA regions are required for the VirF-dependent expression of *virB* [81]. The first one is located in the *virB* promoter (−110 to −80), as expected. The second, however, is located in the coding sequence of the neighboring genes *ipaA* and *acp* (−976 to −402). Binding at both sites may be necessary to completely remove H-NS from the area. In brief, VirF is the master activator of the gene regulatory cascade and it acts primarily by inhibiting H-NS-mediated silencing. This allows VirF to directly control the expression of *icsA* required for cell-to-cell spread, and indirectly control the expression of the T3SS by regulating the expression of VirB.

The second step in the gene regulatory cascade is controlled by VirB. It is a transcriptional activator of the ParB family whose members are mostly DNA partition proteins. Nevertheless, VirB activates the transcription of T3SS-related genes in the entry region and elsewhere in pINV, hence playing the central role in the expression of the T3SS (Figure 3a,b). Its consensus binding sequence is known, although there are conflicting reports on its size [81,82,83,84]. The largest proposed binding site seems to be relevant because its occurrence is proportional to the number of genes regulated by VirB [81]. Furthermore, VirB is involved in two positive feedback loops that stimulate the transcription of its target genes as well as that of *virF* at 37 °C [83]. Interestingly, through interactions with its binding sites, VirB is recruited to distinct cellular foci in a manner similar to related proteins involved in plasmid partitioning [85]. Indeed, a single VirB DNA binding site of 25 base pairs installed on a small plasmid induced GFP-VirB objects visible by light microscopy, suggesting that VirB may form large oligomers on its target gene. Like VirF, VirB probably functions by displacing H-NS from its target genes. It does this likely by forming large oligomers that nucleate from binding sites that can be within a few hundred base pairs from the transcription start sites [71,76,84,86,87,88,89]. In the absence of H-NS, VirB was unable to stimulate transcription. This suggests that it acts as an antisilencing factor to derepress transcription [88]. It has been proposed that VirB relieves H-NS silencing by reducing negative supercoiling of the DNA in the vicinity of its binding sites [90]. In short, VirF together with VirB through the transcription of their target genes at 37 °C ensure that the T3SS is assembled and ready to function only when *Shigella* is in its host. VirB controls the expression of the invasive phenotype through the production of the injectisome parts, chaperones, and early, middle, and late substrates A. It also controls the expression of the third transcriptional activator MxiE [12].

The final step in the gene regulatory cascade is controlled by MxiE. It is a transcriptional activator of the AraC family that controls the expression of genes encoding a subset of the T3SS substrates called the late substrates B [12], also known as second wave effectors. The activity of MxiE is regulated at the post-translational level by the activity of the injectisome (Figure 3a). Prior to secretion, the formation of a 1:1 complex between IpgC and IpaB [91], or with IpaC, and between OspD1 and MxiE [92], prevents MxiE-IpgC-mediated transcriptional activation. By contrast, upon the T3SS-mediated secretion of the antiactivator OspD1 and the anticoactivators IpaB and IpaC, MxiE, in cooperation with its co-activator IpgC, activates the expression of genes encoding late substrates B [12,92,93,94], which are secreted last during host cell invasion. The genes regulated by MxiE and IpgC have a consensus MxiE box in their promoter [95,96]. The binding of a MxiE-IpgC complex to the MxiE box has been inferred from the comparable dampening of gene expression in Δ*mxiE* and Δ*ipgC* strains [94], but has not been demonstrated. On the other hand, the existence of a MxiE-IpgC complex is supported by the co-elution of these proteins from an affinity column [97]. MxiE was thought to activate transcription by promoting the recruitment of the RNA polymerase, similar to known members of the AraC family [98]. However, in *Shigella* expressing a defective H-NS, MxiE is dispensable for the transcription of target genes [99]. This suggests that MxiE may activate transcription by inhibiting H-NS silencing similarly to VirF and VirB. Finally, MxiE and IpgC form a negative feedback loop that represses *virB*, but not *icsA*, in a VirF-dependent manner [81]. Whether, this requires the formation of a tripartite complex between these proteins is unknown. The genes activated by MxiE and IpgC were initially determined from a gene microarray focused on the virulence plasmid [100] (Figure 3b). Several of the genes regulated by MxiE have fascinating enzymatic activities that hijack the host cell immune response and programed cell death pathways [10,101,102,103,104,105,106,107]. Thus, the effectors encoded by MxiE-regulated genes are critical for post-invasion steps of pathogenesis, particularly for evading host defense. In brief, MxiE-mediated transcriptional regulation is critical for controlling the expression of the late substrate B, thereby contributing to the orderly secretion of substrates via the T3SS [12]. 

For years, the only MxiE-regulated chromosomal genes were *ipaH* genes, which have family members in pINV [96]. By contrast, a genome-wide transcriptome analysis of *Shigella* added two new genes to the MxiE regulon [49]. The proteins encoded by these genes are secreted by the T3SS as late substrates B [108]. We named them *icaR* (also known as gem3 or *yjgL*), and *icaT* (also known as gem1 or *yfdF*), which stand for invasion chromosome antigen with homology for a transcription regulator and a transposase, respectively. Interestingly, the appearance of *icaR* and *icaT* likely preceded the acquisition of pINV, as they are found in several *E. coli* phylogroups [108]. An independent genome-wide transcriptome analysis identified *yccE* as a third novel chromosomal gene regulated by MxiE and IpgC [99]. Indeed, *yccE* possessed a MxiE box and is regulated by the activity of the T3SS, similar to *icaR* and *icaT*. In addition, *icaR* (*yjgL*), *icaT* (*yfdF*), and *yccE* were previously reported to be upregulated in the absence of H-NS in *E. coli* [66], thereby supporting the notion that MxiE inhibits H-NS silencing to activate its target genes [99]. Upon re-examination of the raw data in [49], we found a short operon called *yhaBC* that harbors an unstudied MxiE-box that is remarkably similar to those of *yccE*, *icaR*, and *icaT* (Figure 3c). Furthermore, we confirmed that *yccE* and *yhaBC* are present in almost all *E. coli* strains harboring *icaR* and *icaT* [108] (Table A1). Based on this evidence, we speculate that, long before the acquisition of the modern pINV by *Shigella*, the *E. coli* lineage harbored one or more mobile genetic elements carrying these four ancient genes and their transcriptional regulators *mxiE* and *ipgC* or relatives. These four genes were independently transferred to the chromosome at about the same time, and silenced by the loss of their regulators until reactivated by the acquisition of pINV. Given their ancestral nature, these genes are unlikely to be critical for pathogenesis, although this has not been ruled out at the time of writing.

## 5. Chromosome Factors Modulating the Virulence Gene Regulatory Cascade

**Figure 4:**
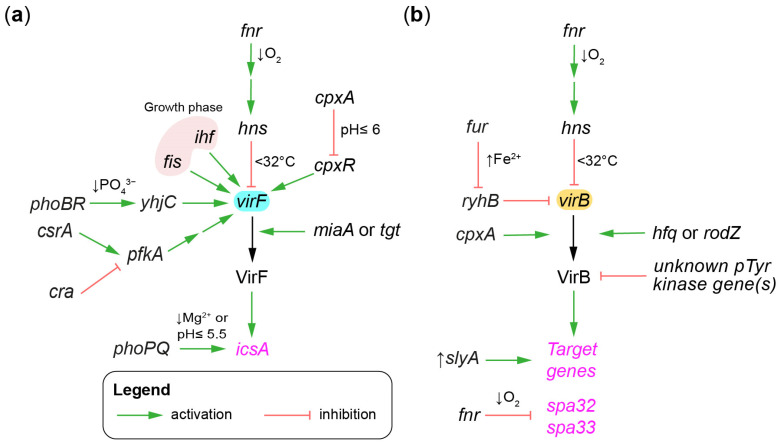
Chromosome genes regulating the gene regulatory cascade. Chromosome genes and environmental cues modulating the expression of: (**a**) *virF*; (**b**) *virB*. The arrows toward *virF* and *virB* indicate transcriptional regulation. The arrows toward VirB indicate post-translational regulation. The arrows toward the black arrow joining *virF*-VirF or *virB*-VirB indicate post-transcriptional or translational regulation. Arrows toward VirB indicate post-translational regulation. The regulators and their terminal targets are written in black and magenta, respectively.

The activity of the virulence gene regulatory cascade is modulated by metabolic or environmental parameters that are sensed by several chromosome-encoded factors. In this section, the factors regulating *virF* and *virB* will be discussed in turn. For example, *virF* is the nexus of several signaling cross-talks involving proteins such as FIS, HIF, MiaA, and TGT, which have been reviewed elsewhere [73] (Figure 4a).

In addition, several two-component systems have been implicated in the adaptation of *Shigella*’s virulence to its environment [109]. For instance, during its journey through the gastrointestinal tract, *Shigella* switches from an acidic environment in the stomach to a near-neutral pH in the terminal ileum and in the descending colon [110]. The two-component system *cpxAR*, which is involved in the membrane stress response, regulates the expression of *virF* in a pH-dependent manner. At physiological pH (7.4), *virF* is well expressed, whereas at acidic pH (6.0), *virF* expression is reduced, resulting in a 10-fold reduction in *S. sonnei* invasiveness [111]. The response regulator CpxR directly binds to the *virF* promoter and is essential for its activity at both pHs [112], whereas the sensor kinase CpxA is only required for acidic pH repression [111]. These findings suggest that CpxR might be activated by another sensor kinase at physiological pH. The elongation factor P regulates this process by stimulating the translation of *cpxA* in *S. flexneri* [113]. Finally, CpxA promotes *virB* expression at the post-transcriptional level through an unknown mechanism [114] (Figure 4b).

PhoBR is a two-component system that is activated by phosphate starvation. PhoB moderately activated the expression of *yhjC* [115], which encodes a LysR transcriptional regulator that activates *virF* expression in *Shigella*. Indeed, YhjC binds directly to the *virF* promoter to activate its expression, and in Δ*yhjC*, the expression of *virF* and of its targets *virB* and *icsA* were downregulated, which dampened the invasiveness of this strain [116].

PhoPQ is a two-component system involved in the adaptation to low Mg^2+^ and the expression of acid resistance genes [117]. A Δ*phoPQ* strain was attenuated in cell invasion assays and in the Sereny test, and was more sensitive to the antimicrobial polymixin B. The expression of 11 genes, including *icsA*, was activated by PhoP, which directly bound their promoter. This two-component system is also involved in the regulation of pINV genes that are unrelated to the T3SS, but nonetheless involved in virulence [51,118].

The glycolysis pathway also modulates the expression of *virF* in *S. flexneri*. Indeed, a strain lacking *pfkA*, which encodes the major phoshphofructokinase, was attenuated due to reduced *virF* expression [119]. Two regulators of the glycolytic pathway, the mRNA binding protein *csrA* and the transcriptional regulator *cra*, also affected the virulence. This study suggested this was through their effect on *pfkA*. The mechanism by which *pfkA* regulates *virF* is currently unknown, but is likely indirect. Interestingly, *csrA* regulates the T3SS in other bacteria [120].

Iron levels in *E. coli* are sensed by the ferric uptake regulator protein (Fur). Iron (II)-bound Fur, also known as holo-Fur, represses most of its target genes [121,122,123], such as the small RNA RyhB. By contrast, when the cytosolic concentration of iron is low, the concentration of RyhB increases, which represses its own target genes [124,125]. In *Shigella*, RyhB binds to a complementary region in the coding sequence of VirB to repress its expression [126,127]. RyhB targets the template strand of VirB [127], suggesting a transcriptional interference mechanism. Therefore, a reduced amount of VirB through the action of RyhB would lead to a lower production of injectisome parts, and consequently to a reduction in the number of injectisomes on the bacterial surface. Indeed, overexpression of RyhB reduced plaque formation by 20-fold in a strain that contained an integral RyhB binding site in *virB*, whereas a strain with a disrupted binding site was unaffected [127]. The RyhB-mediated downregulation of VirB also decreased the expression of *icsP* [128], which is important for the proper function of IcsA in cell-to-cell spreading. Taken together, iron replete conditions enhanced the invasion phenotype by stimulating the T3SS and cell-to-cell spreading. By contrast, the *stx* gene, which encodes the Shiga toxin, is upregulated under iron-deficient conditions in *E. coli* and in *S. dysenteriae* [123,129,130]. This suggests that iron controls the balance between the invasion and Shiga toxin arms of *S. dysenteriae* pathogenesis.

T3SS expression is also repressed under anaerobic conditions via the activation of the fumarate and nitrate reduction regulatory protein (FNR). Two distinct mechanisms have been proposed. First, FNR represses the expression of genes encoding the switch regulator Spa32 and the injectisome part Spa33 by directly binding to their promoters [131]. Second, through an unknown mechanism FNR cascades to enhance the H-NS-mediated silencing of *virF* and *virB*, thereby inducing a broader downregulation of the T3SS than initially hypothesized [132]. The expression of several chromosome genes was also affected by oxygen deprivation in an FNR-dependent or -independent manner. This suggests an adaptation of *Shigella* to hypoxia that extends beyond the T3SS. Whether these adaptations are specific to *Shigella* or shared with *E. coli* has not been investigated to our knowledge.

VirB is also targeted at the post-transcriptional level. For example, the destabilization of the mRNA of *virB* by the RNA chaperone Hfq and the cytoskeletal protein RodZ could contribute to the repression of VirB production, particularly at 30 °C [133,134]. This process would potentially synergize with the H-NS silencing to restrict virulence at ambient temperatures. Several virulence-associated proteins have been identified in the *Shigella* phosphotyrosine proteome [135]. The phosphorylation of VirB on its Tyr100 could inhibit its function. Indeed, the introduction of a phosphomimetic mutation at this position prevented the expression of its target gene *icsP*. The abundance and significance of phospho-VirB and its cognate protein tyrosine kinase are unknown. The consensus sequence of the phosphosites is well conserved [135], suggesting that one or a small group of related kinases may be responsible for most of the phospotyrosines [136]. The significance of this mode of VirB regulation is unknown, but is probably minor compared to its transcriptional regulation.

SlyA is a transcriptional regulator of the MarR/SlyA family that activates the expression of chaperones and acid resistance genes, and represses genes in the histidine biosynthetic pathway. It is negatively autoregulated and positively regulated by PhoP [137]. It is also involved in the regulation of T3SS genes through an unknown mechanism. Indeed, in a Δ*virB* strain, the production of SlyA at supraphysiological levels can rescue the expression of injectisome parts [137]. Notably, SlyA has been hypothesized to derepress H-NS-mediated silencing similarly to VirB [138], thus suggesting a general mechanism by which supraphysiological levels of SlyA could complement Δ*virB*. The significance of SlyA as an alternative counter-silencing factor of the entry region in the context of infection is unknown.

To our knowledge, there are no chromosome-encoded factors, other than H-NS, that modulate the activity of *mxiE* or of its cognate protein. Since the function of MxiE normally occurs during the invasion of host cells, whose environmental conditions cannot be reproduced in vitro, the modulation of this transcriptional activator is difficult to study. It is therefore plausible that such modulatory processes are just waiting to be discovered.

## 6. Conclusions

The evolution of the different *Shigella* subgroups and of EIECs is linked to the acquisition of the invasion plasmid pINV. Although most strains converge towards similar genetic adaptation, such as shared gene attrition, it is reasonable to assume that they may not be at the same stage of coevolution of their chromosome with pINV. The comparative study of carefully selected *Shigella* and EIEC strains belonging to different phylogroups could be harnessed to reconstruct the evolutionary interactions between their genome and the pathogenic lifestyle conferred by pINV. A contrasting model of interest is *Yersinia*, which has its own T3SS-encoding plasmid and adopts an extracellular pathogenic lifestyle different from that of *Shigella*. Perhaps such approaches could be used to create predictive models of the future evolution of *Shigella* and *Yersinia* pathogenesis. Due to its recent emergence, *Shigella* pathogenesis can be described as an intermediate state between commensalism and obligate pathogenesis. Interestingly, the metastability of its virulence is mirrored by that of pINV, which is less stable in *S. sonnei* than in *S. flexneri*. The genetic interplay between the pINV and the chromosome extends to the expression of virulence traits. Indeed, the master silencer H-NS and a plethora of secondary regulators encoded in the chromosome modulate the virulence gene regulatory cascade centered around the pINV transcriptional activators VirF, VirB, and MxiE. This cross-talk is indeed surprisingly rich given the short evolutionary timeline of *Shigella*. Furthermore, the MxiE-regulated chromosomal genes *icaR*, *icaT*, *yccE*, and the poorly characterized *yhaBC* operon are conserved across *E. coli* phylogroups. Taken together, these observations support the idea that the last common ancestor of *Shigella* and several *E. coli* had a long association with pINV, its ancestors, or related plasmids. This provides further evidence that *E. coli*, including *Shigella* spp., have walked the fine line between commensalism and pathogenesis throughout much of their evolution. Future advances promise to deepen our understanding of the fluidity between commensalism and pathogenesis in *E. coli* and *Shigella* spp. Finally, the detailed understanding of the virulence gene regulatory cascade and its regulators provides opportunities to develop antivirulence therapeutic strategies. Indeed, small molecules that suppress the expression of the T3SS could be useful to contain *Shigella* outbreaks and to replace antibiotics.

## Figures and Tables

**Table 1:** Distribution of *Shigella* strains in *E. coli* phylogroups ^1^.

Phylogroups	Total Sequences	*Shigella* Sequences	Percentage *Shigella* in Group (%)	Percentage of Total *Shigella* (%)
All	10,667	1283	12.0	100
Shig1	177	177	100	70.1
Shig2	899	899	100	13.8
B1	2960	140	4.73	10.9
A	2232	44	1.97	3.43
E1	279	9	3.23	0.70
D3	177	7	3.95	0.55
D2	177	4	2.26	0.31
F	199	2	1.01	0.16
B2-2	1367	1	0.07	0.08
G	96	0	0	0
D1	D1	0	0	0
C	540	0	0	0
B2-1	541	0	0	0

^1^ The data in this table are from the Supplementary Information from Abram et al. [37].

## Data Availability

Not applicable.

## Appendix A

**Table A1:** Occurrence of *yccE* and *yhaBC* in strains in which *icaR* and *icaT* presence was previously investigated [109] ^1^.

Phylogroups	Strains Name	Accession Number	*yccE*	*yhaBC*
*Shigella*	*S. flexneri* 5a str. M90T	NZ_CM001474.1	Yes	Yes
	*S. flexneri* 2a str. 301	NC_00337.2	Yes	Yes
	*S. boydii* Sb227	NC_007613.1	Yes	Yes
	*S. sonnei* strain SE6-1	NZ_CP055292.1	Yes	Yes
	*S. sonnei* Ss046	CP000038.1	Yes	No ^2^
	*S. dysenteriae* Sd197	NC_007606.1	Yes	No ^2^
A	*E. coli* str. K-12 substr. MG1655	U00096.3	Yes	Yes
	*E. coli* strain NCTC86	NZ_CP019778.1	Yes	Yes
	*E. coli* ETEC H10407	NC_017633.1	Yes	Yes
	*E. coli* HS	NC_009800.1	Yes	Yes
B1	*E. coli* O103:H2 str. 12009	NC_013353.1	Yes	Yes
	*E. coli* 55989	NC_011748.1	Yes	Yes
	*E. coli* W	NC_017635.1	Yes	Yes
	*E. coli* O26: H11 str. 11368	NC_013361.1	Yes	Yes
B2	*E. coli* O127:H6 str. E2348/69	NC_011601.1	No	No
	*E. coli* 536	NC_008253.1	No	No
	*E. coli* SE15	NC_013654.1	No	No
	*E. coli* ABU 83972	NC_017631.1	No	No
	*E. coli* CFT073	NZ_CP051263.1	No	No
D	*E. coli* 042	NC_017626.1	Yes	Yes
	*E. coli* UMN026	NC_011751.1	Yes	Yes
	*E. coli* SMS-3-5	NC_010498.1	Yes	Yes
	*E. coli* IAI39	NC_011750.1	Yes	Yes
E	*E. coli* O157:H7 strain ATCC43888	NZ_CP041623.1	Yes	Yes
	*E. coli* O157:H7 str. Sakai	NC_002695.1	Yes	Yes
	*E. coli* O55:H7 str. CB9615	NC_013941.1	Yes	Yes
	*E. coli* O157:H7 str. EDL933	NZ_CP008957.1	Yes	Yes

^1^ *yccE* and *yhaBC* are present in the same strains as *icaR* and *icaT*. One main exception: a partly disrupted *icaR* was observed in *E. coli* O127:H6 str. E2348/69, whereas *icaT*, *yccE*, and *yhaBC* were absent. *icaR* and *icaT* were also pseudogenes in *S. sonnei* Ss046 and *S. dysenteriae* Sd197 [109]. *yhaC* has a premature stop codon in *S. flexneri* 5a str. M90T. The BLAST reported in this table were performed with this truncated version; we have not investigated whether *yhaC* is integral in the other strains. ^2^ *yhaBC* is present as a pseudogene in these *Shigella* strains, similar to *icaT* [109].

## References

[1] Hu B., Morado D.R., Margolin W., Rohde J.R., Arizmendi O., Picking W.L., Picking W.D., Liu J. (2015). Visualization of the Type III Secretion Sorting Platform of *Shigella flexneri*. Proc. Natl. Acad. Sci. USA.

[2] Tachiyama S., Chang Y., Muthuramalingam M., Hu B., Barta M.L., Picking W.L., Liu J., Picking W.D. (2019). The Cytoplasmic Domain of MxiG Interacts with MxiK and Directs Assembly of the Sorting Platform in the *Shigella* Type III Secretion System. J. Biol. Chem..

[3] Hu B., Lara-Tejero M., Kong Q., Galán J.E., Liu J. (2017). In Situ Molecular Architecture of the *Salmonella* Type III Secretion Machine. Cell.

[4] Hu J., Worrall L.J., Vuckovic M., Hong C., Deng W., Atkinson C.E., Brett Finlay B., Yu Z., Strynadka N.C.J. (2019). T3S Injectisome Needle Complex Structures in Four Distinct States Reveal the Basis of Membrane Coupling and Assembly. Nat. Microbiol..

[5] Guo E.Z., Galán J.E. (2021). Cryo-EM Structure of the Needle Filament Tip Complex of the *Salmonella* Type III Secretion Injectisome. Proc. Natl. Acad. Sci. USA.

[6] Park D., Lara-Tejero M., Waxham M.N., Li W., Hu B., Galán J.E., Liu J. (2018). Visualization of the Type III Secretion Mediated *Salmonella*-Host Cell Interface Using Cryo-Electron Tomography. eLife.

[7] Deng W., Marshall N.C., Rowland J.L., McCoy J.M., Worrall L.J., Santos A.S., Strynadka N.C.J., Finlay B.B. (2017). Assembly, Structure, Function and Regulation of Type III Secretion Systems. Nat. Rev. Microbiol..

[8] Lara-Tejero M., Galán J.E. (2019). The Injectisome, a Complex Nanomachine for Protein Injection into Mammalian Cells. EcoSal Plus.

[9] Hu J., Worrall L.J., Strynadka N.C. (2020). Towards Capture of Dynamic Assembly and Action of the T3SS at near Atomic Resolution. Curr. Opin. Struct. Biol..

[10] Schnupf P., Sansonetti P.J. (2019). *Shigella* Pathogenesis: New Insights through Advanced Methodologies. Microbiol. Spectr..

[11] Kang E., Crouse A., Chevallier L., Pontier S.M., Alzahrani A., Silué N., Campbell-Valois F.-X., Montagutelli X., Gruenheid S., Malo D. (2018). Enterobacteria and Host Resistance to Infection. Mamm. Genome.

[12] Bajunaid W., Haidar-Ahmad N., Kottarampatel A.H., Manigat F.O., Silué N., Tchagang C.F., Tomaro K., Campbell-Valois F.-X. (2020). The T3ss of *Shigella*: Expression, Structure, Function, and Role in Vacuole Escape. Microorganisms.

[13] Lampel K.A., Formal S.B., Maurelli A.T. (2018). A Brief History of *Shigella*. EcoSal Plus.

[14] Lan R., Reeves P.R. (2002). Escherichia Coli in Disguise: Molecular Origins of *Shigella*. Microbes Infect..

[15] Watkins L.K.F., Appiah G.D. Shigellosis—Chapter 4—2020 Yellow Book|Travelers’ Health|CDC. https://wwwnc.cdc.gov/travel/yellowbook/2020/travel-related-infectious-diseases/shigellosis.

[16] Kotloff K.L., Riddle M.S., Platts-Mills J.A., Pavlinac P., Zaidi A.K.M. (2018). Shigellosis. Lancet.

[17] GBD 2016 Diarrhoeal Disease Collaborators (2018). Estimates of the Global, Regional, and National Morbidity, Mortality, and Aetiologies of Diarrhoea in 195 Countries: A Systematic Analysis for the Global Burden of Disease Study 2016. Lancet Infect. Dis..

[18] Thompson C.N., Duy P.T., Baker S. (2015). The Rising Dominance of *Shigella sonnei*: An Intercontinental Shift in the Etiology of Bacillary Dysentery. PLoS Negl. Trop. Dis..

[19] Kothary M.H., Babu U.S. (2001). Infective Dose of Foodborne Pathogens in Volunteers: A Review. J. Food Saf..

[20] World Health Organization (2017). List of Antibiotic Resistant Bacteria to Guide Research, Discovery and Development of New Antibiotics.

[21] Iredell J., Brown J., Tagg K. (2016). Antibiotic Resistance in Enterobacteriaceae: Mechanisms and Clinical Implications. BMJ.

[22] Lynch J.P., Clark N.M., Zhanel G.G. (2021). Escalating Antimicrobial Resistance among Enterobacteriaceae: Focus on Carbapenemases. Expert Opin. Pharmacother..

[23] Antimicrobial Resistance Collaborators (2022). Global Burden of Bacterial Antimicrobial Resistance in 2019: A Systematic Analysis. Lancet.

[24] Center for Disease Control and Prevention (USA) (2019). Antibiotic Resistance Threats in the United States.

[25] Baker S., Scott T.A. (2023). Antimicrobial-Resistant *Shigella*: Where Do We Go Next?. Nat. Rev. Microbiol..

[26] Lefèvre S., Njamkepo E., Feldman S., Ruckly C., Carle I., Lejay-Collin M., Fabre L., Yassine I., Frézal L., Pardos de la Gandara M. (2023). Rapid Emergence of Extensively Drug-Resistant *Shigella sonnei* in France. Nat. Commun..

[27] Mason L.C.E., Greig D.R., Cowley L.A., Partridge S.R., Martinez E., Blackwell G.A., Chong C.E., De Silva P.M., Bengtsson R.J., Draper J.L. (2023). The Evolution and International Spread of Extensively Drug Resistant *Shigella sonnei*. Nat. Commun..

[28] Pupo G.M., Lan R., Reeves P.R. (2000). Multiple Independent Origins of *Shigella* Clones of *Escherichia coli* and Convergent Evolution of Many of Their Characteristics. Proc. Natl. Acad. Sci. USA.

[29] The H.C., Thanh D.P., Holt K.E., Thomson N.R., Baker S. (2016). The Genomic Signatures of *Shigella* Evolution, Adaptation and Geographical Spread. Nat. Rev. Microbiol..

[30] Holt K.E., Baker S., Weill F.-X., Holmes E.C., Kitchen A., Yu J., Sangal V., Brown D.J., Coia J.E., Kim D.W. (2012). *Shigella sonnei* Genome Sequencing and Phylogenetic Analysis Indicate Recent Global Dissemination from Europe. Nat. Genet..

[31] Njamkepo E., Fawal N., Tran-Dien A., Hawkey J., Strockbine N., Jenkins C., Talukder K.A., Bercion R., Kuleshov K., Kolínská R. (2016). Global Phylogeography and Evolutionary History of *Shigella dysenteriae* Type 1. Nat. Microbiol..

[32] Connor T.R., Barker C.R., Baker K.S., Weill F.-X., Talukder K.A., Smith A.M., Baker S., Gouali M., Pham Thanh D., Jahan Azmi I. (2015). Species-Wide Whole Genome Sequencing Reveals Historical Global Spread and Recent Local Persistence in *Shigella flexneri*. eLife.

[33] Maurelli A.T., Fernández R.E., Bloch C.A., Rode C.K., Fasano A. (1998). “Black Holes” and Bacterial Pathogenicity: A Large Genomic Deletion That Enhances the Virulence of *Shigella* spp. and Enteroinvasive *Escherichia coli*. Proc. Natl. Acad. Sci. USA.

[34] Maurelli A.T. (2007). Black holes, antivirulence genes, and gene inactivation in the evolution of bacterial pathogens. FEMS Microbiol. Lett..

[35] Hawkey J., Monk J.M., Billman-Jacobe H., Palsson B., Holt K.E. (2020). Impact of Insertion Sequences on Convergent Evolution of *Shigella* Species. PLoS Genet..

[36] Escobar-Páramo P., Giudicelli C., Parsot C., Denamur E. (2003). The Evolutionary History of *Shigella* and Enteroinvasive *Escherichia coli* Revised. J. Mol. Evol..

[37] Abram K., Udaondo Z., Bleker C., Wanchai V., Wassenaar T.M., Robeson M.S., Ussery D.W. (2021). Mash-Based Analyses of Escherichia Coli Genomes Reveal 14 Distinct Phylogroups. Commun. Biol..

[38] Touchon M., Perrin A., de Sousa J.A.M., Vangchhia B., Burn S., O’Brien C.L., Denamur E., Gordon D., Rocha E.P. (2020). Phylogenetic Background and Habitat Drive the Genetic Diversification of *Escherichia coli*. PLoS Genet..

[39] Gonzalez-Alba J.M., Baquero F., Cantón R., Galán J.C. (2019). Stratified Reconstruction of Ancestral *Escherichia coli* Diversification. BMC Genom..

[40] Buchrieser C., Glaser P., Rusniok C., Nedjari H., D’Hauteville H., Kunst F., Sansonetti P., Parsot C. (2000). The Virulence Plasmid PWR100 and the Repertoire of Proteins Secreted by the Type III Secretion Apparatus of *Shigella flexneri*. Mol. Microbiol..

[41] Cervantes-Rivera R., Tronnet S., Puhar A. (2020). Complete Genome Sequence and Annotation of the Laboratory Reference Strain *Shigella flexneri* Serotype 5a M90T and Genome-Wide Transcriptional Start Site Determination. BMC Genom..

[42] Pasqua M., Michelacci V., Di Martino M.L., Tozzoli R., Grossi M., Colonna B., Morabito S., Prosseda G. (2017). The Intriguing Evolutionary Journey of Enteroinvasive *E. coli* (EIEC) toward Pathogenicity. Front. Microbiol..

[43] Yang F., Yang J., Zhang X., Chen L., Jiang Y., Yan Y., Tang X., Wang J., Xiong Z., Dong J. (2005). Genome Dynamics and Diversity of *Shigella* Species, the Etiologic Agents of Bacillary Dysentery. Nucleic Acids Res..

[44] Sims G.E., Kim S.-H. (2011). Whole-Genome Phylogeny of *Escherichia coli*/*Shigella* Group by Feature Frequency Profiles (FFPs). Proc. Natl. Acad. Sci. USA.

[45] Campbell-Valois F.-X., Pontier S.M. (2016). Implications of Spatiotemporal Regulation of *Shigella flexneri* Type Three Secretion Activity on Effector Functions: Think Globally, Act Locally. Front. Cell. Infect. Microbiol..

[46] Izoré T., Job V., Dessen A. (2011). Biogenesis, Regulation, and Targeting of the Type III Secretion System. Structure.

[47] Abby S.S., Cury J., Guglielmini J., Néron B., Touchon M., Rocha E.P.C. (2016). Identification of Protein Secretion Systems in Bacterial Genomes. Sci. Rep..

[48] Stevens J.M., Galyov E.E., Stevens M.P. (2006). Actin-Dependent Movement of Bacterial Pathogens. Nat. Rev. Microbiol..

[49] Silué N., Marcantonio E., Campbell-Valois F.-X. (2020). RNA-Seq Analysis of the T3SA Regulon in *Shigella flexneri* Reveals Two New Chromosomal Genes Upregulated in the on-State. Methods.

[50] D’Hauteville H., Khan S., Maskell D.J., Kussak A., Weintraub A., Mathison J., Ulevitch R.J., Wuscher N., Parsot C., Sansonetti P.J. (2002). Two MsbB Genes Encoding Maximal Acylation of Lipid A Are Required for Invasive *Shigella flexneri* to Mediate Inflammatory Rupture and Destruction of the Intestinal Epithelium. J. Immunol..

[51] Goldman S.R., Tu Y., Goldberg M.B. (2008). Differential Regulation by Magnesium of the Two MsbB Paralogs of *Shigella flexneri*. J. Bacteriol..

[52] Sidik S., Kottwitz H., Benjamin J., Ryu J., Jarrar A., Garduno R., Rohde J.R. (2014). A *Shigella flexneri* Virulence Plasmid Encoded Factor Controls Production of Outer Membrane Vesicles. G3 Genes Genomes Genet..

[53] Meza-Segura M., Birtley J.R., Maldonado-Contreras A., Mueller C., Simin K.J., Stern L.J., McCormick B.A. (2021). SepA Enhances *Shigella* Invasion of Epithelial Cells by Degrading Alpha-1 Antitrypsin and Producing a Neutrophil Chemoattractant. mBio.

[54] Lan R., Lumb B., Ryan D., Reeves P.R. (2001). Molecular Evolution of Large Virulence Plasmid in *Shigella* Clones and Enteroinvasive *Escherichia coli*. Infect. Immun..

[55] Maeda S., Ito M., Ando T., Ishimoto Y., Fujisawa Y., Takahashi H., Matsuda A., Sawamura A., Kato S. (2006). Horizontal Transfer of Nonconjugative Plasmids in a Colony Biofilm of *Escherichia coli*. FEMS Microbiol. Lett..

[56] Schuch R., Maurelli A.T. (1997). Virulence Plasmid Instability in *Shigella flexneri* 2a Is Induced by Virulence Gene Expression. Infect. Immun..

[57] Pilla G., Tang C.M. (2018). Going around in Circles: Virulence Plasmids in Enteric Pathogens. Nat. Rev. Microbiol..

[58] Martyn J.E., Pilla G., Hollingshead S., Winther K.S., Lea S., McVicker G., Tang C.M. (2022). Maintenance of the *Shigella sonnei* Virulence Plasmid Is Dependent on Its Repertoire and Amino Acid Sequence of Toxin-Antitoxin Systems. J. Bacteriol..

[59] McVicker G., Hollingshead S., Pilla G., Tang C.M. (2019). Maintenance of the Virulence Plasmid in *Shigella flexneri* Is Influenced by Lon and Two Functional Partitioning Systems. Mol. Microbiol..

[60] McVicker G., Tang C.M. (2016). Deletion of Toxin-Antitoxin Systems in the Evolution of *Shigella sonnei* as a Host-Adapted Pathogen. Nat. Microbiol..

[61] Fraikin N., Goormaghtigh F., Van Melderen L. (2020). Type II Toxin-Antitoxin Systems: Evolution and Revolutions. J. Bacteriol..

[62] Kamruzzaman M., Wu A.Y., Iredell J.R. (2021). Biological Functions of Type II Toxin-Antitoxin Systems in Bacteria. Microorganisms.

[63] Pilla G., McVicker G., Tang C.M. (2017). Genetic Plasticity of the *Shigella* Virulence Plasmid Is Mediated by Intra- and Inter-Molecular Events between Insertion Sequences. PLoS Genet..

[64] Pilla G., Arcari G., Tang C.M., Carattoli A. (2022). Virulence Plasmid PINV as a Genetic Signature for *Shigella flexneri* Phylogeny. Microb. Genom..

[65] Picker M.A., Wing H.J. (2016). H-NS, Its Family Members and Their Regulation of Virulence Genes in *Shigella* Species. Genes.

[66] Lamberte L.E., Baniulyte G., Singh S.S., Stringer A.M., Bonocora R.P., Stracy M., Kapanidis A.N., Wade J.T., Grainger D.C. (2017). Horizontally Acquired AT-Rich Genes in Escherichia Coli Cause Toxicity by Sequestering RNA Polymerase. Nat. Microbiol..

[67] Riccardi E., van Mastbergen E.C., Navarre W.W., Vreede J. (2019). Predicting the Mechanism and Rate of H-NS Binding to AT-Rich DNA. PLoS Comput. Biol..

[68] Shen B.A., Hustmyer C.M., Roston D., Wolfe M.B., Landick R. (2022). Bacterial H-NS Contacts DNA at the Same Irregularly Spaced Sites in Both Bridged and Hemi-Sequestered Linear Filaments. iScience.

[69] Liu Y., Chen H., Kenney L.J., Yan J. (2010). A Divalent Switch Drives H-NS/DNA-Binding Conformations between Stiffening and Bridging Modes. Genes Dev..

[70] Tobe T., Yoshikawa M., Mizuno T., Sasakawa C. (1993). Transcriptional Control of the Invasion Regulatory Gene VirB of *Shigella flexneri*: Activation by VirF and Repression by H-NS. J. Bacteriol..

[71] Beloin C., Dorman C.J. (2003). An Extended Role for the Nucleoid Structuring Protein H-NS in the Virulence Gene Regulatory Cascade of *Shigella flexneri*. Mol. Microbiol..

[72] Falconi M., Colonna B., Prosseda G., Micheli G., Gualerzi C.O. (1998). Thermoregulation of *Shigella* and Escherichia *coli* EIEC Pathogenicity. A Temperature-Dependent Structural Transition of DNA Modulates Accessibility of VirF Promoter to Transcriptional Repressor H-NS. EMBO J..

[73] Di Martino M.L., Falconi M., Micheli G., Colonna B., Prosseda G. (2016). The Multifaceted Activity of the VirF Regulatory Protein in the *Shigella* Lifestyle. Front. Mol. Biosci..

[74] Di Martino M.L., Romilly C., Wagner E.G.H., Colonna B., Prosseda G. (2016). One Gene and Two Proteins: A Leaderless MRNA Supports the Translation of a Shorter Form of the *Shigella* VirF Regulator. mBio.

[75] Skovajsová E., Colonna B., Prosseda G., Sellin M.E., Di Martino M.L. (2022). The VirF21:VirF30 Protein Ratio Is Affected by Temperature and Impacts *Shigella flexneri* Host Cell Invasion. FEMS Microbiol. Lett..

[76] Wing H.J., Yan A.W., Goldman S.R., Goldberg M.B. (2004). Regulation of IcsP, the Outer Membrane Protease of the *Shigella* Actin Tail Assembly Protein IcsA, by Virulence Plasmid Regulators VirF and VirB. J. Bacteriol..

[77] Tran C.N., Giangrossi M., Prosseda G., Brandi A., Di Martino M.L., Colonna B., Falconi M. (2011). A Multifactor Regulatory Circuit Involving H-NS, VirF and an Antisense RNA Modulates Transcription of the Virulence Gene IcsA of *Shigella flexneri*. Nucleic Acids Res..

[78] Giangrossi M., Giuliodori A.M., Tran C.N., Amici A., Marchini C., Falconi M. (2017). VirF Relieves the Transcriptional Attenuation of the Virulence Gene IcsA of *Shigella flexneri* Affecting the IcsA mRNA-RnaG Complex Formation. Front. Microbiol..

[79] Ragazzone N.J., Dow G.T., Garcia G.A. (2022). Elucidation of Key Interactions between VirF and the VirB Promoter in *Shigella flexneri* Using *E. coli* MarA- and GadX-Based Homology Models and In Vitro Analysis of the DNA-Binding Domains of VirF and MarA. J. Bacteriol..

[80] Dow G.T., Young A.M., Garcia G.A. (2023). Elucidation of the DNA-Binding Activity of VirF from *Shigella flexneri* for the IcsA and RnaG Promoters and Characterization of the N-Terminal Domain To Identify Residues Crucial for Dimerization. J. Bacteriol..

[81] McKenna J.A., Karney M.M.A., Chan D.K., Weatherspoon-Griffin N., Becerra Larios B., Pilonieta M.C., Munson G.P., Wing H.J. (2022). The AraC/XylS Protein MxiE and Its Coregulator IpgC Control a Negative Feedback Loop in the Transcriptional Cascade That Regulates Type III Secretion in *Shigella flexneri*. J. Bacteriol..

[82] Taniya T., Mitobe J., Nakayama S., Mingshan Q., Okuda K., Watanabe H. (2003). Determination of the InvE Binding Site Required for Expression of IpaB of the *Shigella sonnei* Virulence Plasmid: Involvement of a ParB BoxA-like Sequence. J. Bacteriol..

[83] Kane K.A., Dorman C.J. (2012). VirB-Mediated Positive Feedback Control of the Virulence Gene Regulatory Cascade of *Shigella flexneri*. J. Bacteriol..

[84] Weatherspoon-Griffin N., Picker M.A., Pew K.L., Park H.S., Ginete D.R., Karney M.M., Usufzy P., Castellanos M.I., Duhart J.C., Harrison D.J. (2018). Insights into Transcriptional Silencing and Anti-Silencing in *Shigella flexneri*: A Detailed Molecular Analysis of the IcsP Virulence Locus. Mol. Microbiol..

[85] Socea J.N., Bowman G.R., Wing H.J. (2021). VirB, a Key Transcriptional Regulator of Virulence Plasmid Genes in *Shigella flexneri*, Forms DNA-Binding Site Dependent Foci in the Bacterial Cytoplasm. J. Bacteriol..

[86] Karney M.M., McKenna J.A., Weatherspoon-Griffin N., Karabachev A.D., Millar M.E., Potocek E.A., Wing H.J. (2019). Investigating the DNA-Binding Site for VirB, a Key Transcriptional Regulator of *Shigella* Virulence Genes, Using an In Vivo Binding Tool. Genes.

[87] Basta D.W., Pew K.L., Immak J.A., Park H.S., Picker M.A., Wigley A.F., Hensley C.T., Pearson J.S., Hartland E.L., Wing H.J. (2013). Characterization of the OspZ Promoter in *Shigella flexneri* and Its Regulation by VirB and H-NS. J. Bacteriol..

[88] Turner E.C., Dorman C.J. (2007). H-NS Antagonism in *Shigella flexneri* by VirB, a Virulence Gene Transcription Regulator That Is Closely Related to Plasmid Partition Factors. J. Bacteriol..

[89] McKenna J.A., Wing H.J. (2020). The Antiactivator of Type III Secretion, OspD1, Is Transcriptionally Regulated by VirB and H-NS from Remote Sequences in *Shigella flexneri*. J. Bacteriol..

[90] Picker M.A., Karney M.M.A., Gerson T.M., Karabachev A.D., Duhart J.C., McKenna J.A., Wing H.J. (2023). Localized Modulation of DNA Supercoiling, Triggered by the *Shigella* Anti-Silencer VirB, Is Sufficient to Relieve H-NS-Mediated Silencing. Nucleic Acids Res..

[91] Ferrari M.L., Charova S.N., Sansonetti P.J., Mylonas E., Gazi A.D. (2021). Structural Insights of *Shigella* Translocator IpaB and Its Chaperone IpgC in Solution. Front. Cell. Infect. Microbiol..

[92] Parsot C., Ageron E., Penno C., Mavris M., Jamoussi K., d’Hauteville H., Sansonetti P., Demers B. (2005). A Secreted Anti-Activator, OspD1, and Its Chaperone, Spa15, Are Involved in the Control of Transcription by the Type III Secretion Apparatus Activity in *Shigella flexneri*. Mol. Microbiol..

[93] Kane C.D., Schuch R., Day W.A.J., Maurelli A.T. (2002). MxiE Regulates Intracellular Expression of Factors Secreted by the *Shigella flexneri* 2a Type III Secretion System. J. Bacteriol..

[94] Mavris M., Page A.L., Tournebize R., Demers B., Sansonetti P., Parsot C. (2002). Regulation of Transcription by the Activity of the *Shigella flexneri* Type III Secretion Apparatus. Mol. Microbiol..

[95] Mavris M., Sansonetti P.J., Parsot C. (2002). Identification of the Cis-Acting Site Involved in Activation of Promoters Regulated by Activity of the Type III Secretion Apparatus in *Shigella flexneri*. J. Bacteriol..

[96] Bongrand C., Sansonetti P.J., Parsot C. (2012). Characterization of the Promoter, MxiE Box and 5′ UTR of Genes Controlled by the Activity of the Type III Secretion Apparatus in *Shigella flexneri*. PLoS ONE.

[97] Pilonieta M.C., Munson G.P. (2008). The Chaperone IpgC Copurifies with the Virulence Regulator MxiE. J. Bacteriol..

[98] Martin R.G., Rosner J.L. (2001). The AraC Transcriptional Activators. Curr. Opin. Microbiol..

[99] Hall C.P., Jadeja N.B., Sebeck N., Agaisse H. (2022). Characterization of MxiE- and H-NS-Dependent Expression of IpaH7.8, OspC1, YccE, and YfdF in *Shigella flexneri*. mSphere.

[100] Gall T.L., Mavris M., Martino M.C., Bernardini M.L., Denamur E., Parsot C. (2005). Analysis of Virulence Plasmid Gene Expression Defines Three Classes of Effectors in the Type III Secretion System of *Shigella flexneri*. Microbiology.

[101] Wandel M.P., Pathe C., Werner E.I., Ellison C.J., Boyle K.B., von der Malsburg A., Rohde J., Randow F. (2017). GBPs Inhibit Motility of *Shigella flexneri* but Are Targeted for Degradation by the Bacterial Ubiquitin Ligase IpaH9.8. Cell Host Microbe.

[102] Li P., Jiang W., Yu Q., Liu W., Zhou P., Li J., Xu J., Xu B., Wang F., Shao F. (2017). Ubiquitination and Degradation of GBPs by a *Shigella* Effector to Suppress Host Defence. Nature.

[103] Goers L., Kim K., Stedman T.C., Canning P.J., Mou X., Ernst N.H., Coers J., Lesser C.F. (2023). *Shigella* IpaH9.8 Limits GBP1-Dependent LPS Release from Intracytosolic Bacteria to Suppress Caspase-4 Activation. Proc. Natl. Acad. Sci. USA.

[104] Luchetti G., Roncaioli J.L., Chavez R.A., Schubert A.F., Kofoed E.M., Reja R., Cheung T.K., Liang Y., Webster J.D., Lehoux I. (2021). *Shigella* Ubiquitin Ligase IpaH7.8 Targets Gasdermin D for Degradation to Prevent Pyroptosis and Enable Infection. Cell Host Microbe.

[105] Hansen J.M., de Jong M.F., Wu Q., Zhang L.-S., Heisler D.B., Alto L.T., Alto N.M. (2021). Pathogenic Ubiquitination of GSDMB Inhibits NK Cell Bactericidal Functions. Cell.

[106] Li Z., Liu W., Fu J., Cheng S., Xu Y., Wang Z., Liu X., Shi X., Liu Y., Qi X. (2021). *Shigella* Evades Pyroptosis by Arginine ADP-Riboxanation of Caspase-11. Nature.

[107] Alphonse N., Wanford J.J., Voak A.A., Gay J., Venkhaya S., Burroughs O., Mathew S., Lee T., Evans S.L., Zhao W. (2022). A Family of Conserved Bacterial Virulence Factors Dampens Interferon Responses by Blocking Calcium Signaling. Cell.

[108] Silué N., Campbell-Valois F.-X. (2022). *IcaR* and *IcaT* Are Ancient Chromosome Genes Encoding Substrates of the Type III Secretion Apparatus in *Shigella flexneri*. mSphere.

[109] Pasqua M., Coluccia M., Eguchi Y., Okajima T., Grossi M., Prosseda G., Utsumi R., Colonna B. (2022). Roles of Two-Component Signal Transduction Systems in *Shigella* Virulence. Biomolecules.

[110] Fallingborg J. (1999). Intraluminal PH of the Human Gastrointestinal Tract. Dan. Med. Bull..

[111] Nakayama S., Watanabe H. (1995). Involvement of CpxA, a Sensor of a Two-Component Regulatory System, in the PH-Dependent Regulation of Expression of *Shigella sonnei* VirF Gene. J. Bacteriol..

[112] Nakayama S., Watanabe H. (1998). Identification of CpxR as a Positive Regulator Essential for Expression of the *Shigella sonnei* VirF Gene. J. Bacteriol..

[113] Marman H.E., Mey A.R., Payne S.M. (2014). Elongation Factor P and Modifying Enzyme PoxA Are Necessary for Virulence of *Shigella flexneri*. Infect. Immun..

[114] Mitobe J., Arakawa E., Watanabe H. (2005). A Sensor of the Two-Component System CpxA Affects Expression of the Type III Secretion System through Posttranscriptional Processing of InvE. J. Bacteriol..

[115] Yang C., Huang T.-W., Wen S.-Y., Chang C.-Y., Tsai S.-F., Wu W.-F., Chang C.-H. (2012). Genome-Wide PhoB Binding and Gene Expression Profiles Reveal the Hierarchical Gene Regulatory Network of Phosphate Starvation in *Escherichia coli*. PLoS ONE.

[116] Li W., Jiang L., Liu X., Guo R., Ma S., Wang J., Ma S., Li S., Li H. (2021). YhjC Is a Novel Transcriptional Regulator Required for *Shigella flexneri* Virulence. Virulence.

[117] Lin Z., Cai X., Chen M., Ye L., Wu Y., Wang X., Lv Z., Shang Y., Qu D. (2017). Virulence and Stress Responses of *Shigella flexneri* Regulated by PhoP/PhoQ. Front. Microbiol..

[118] Kaoukab-Raji A., Biskri L., Bernardini M.-L., Allaoui A. (2012). Characterization of SfPgdA, a *Shigella flexneri* Peptidoglycan Deacetylase Required for Bacterial Persistence within Polymorphonuclear Neutrophils. Microbes Infect..

[119] Gore A.L., Payne S.M. (2010). CsrA and Cra Influence *Shigella flexneri* Pathogenesis. Infect. Immun..

[120] Kusmierek M., Dersch P. (2018). Regulation of Host–Pathogen Interactions via the Post-Transcriptional Csr/Rsm System. Curr. Opin. Microbiol..

[121] Seo S.W., Kim D., Latif H., O’Brien E.J., Szubin R., Palsson B.O. (2014). Deciphering Fur Transcriptional Regulatory Network Highlights Its Complex Role beyond Iron Metabolism in *Escherichia coli*. Nat. Commun..

[122] Troxell B., Hassan H.M. (2013). Transcriptional Regulation by Ferric Uptake Regulator (Fur) in Pathogenic Bacteria. Front. Cell. Infect. Microbiol..

[123] Mey A.R., Gómez-Garzón C., Payne S.M. (2021). Iron Transport and Metabolism in *Escherichia*, *Shigella*, and *Salmonella*. EcoSal Plus.

[124] Massé E., Gottesman S. (2002). A Small RNA Regulates the Expression of Genes Involved in Iron Metabolism in *Escherichia coli*. Proc. Natl. Acad. Sci. USA.

[125] Massé E., Vanderpool C.K., Gottesman S. (2005). Effect of RyhB Small RNA on Global Iron Use in *Escherichia coli*. J. Bacteriol..

[126] Murphy E.R., Payne S.M. (2007). RyhB, an Iron-Responsive Small RNA Molecule, Regulates *Shigella dysenteriae* Virulence. Infect. Immun..

[127] Broach W.H., Egan N., Wing H.J., Payne S.M., Murphy E.R. (2012). VirF-Independent Regulation of *Shigella* VirB Transcription Is Mediated by the Small RNA RyhB. PLoS ONE.

[128] Africa L.A.A., Murphy E.R., Egan N.R., Wigley A.F., Wing H.J. (2011). The Iron-Responsive Fur/RyhB Regulatory Cascade Modulates the *Shigella* Outer Membrane Protease IcsP. Infect. Immun..

[129] Calderwood S.B., Mekalanos J.J. (1987). Iron Regulation of Shiga-like Toxin Expression in *Escherichia coli* Is Mediated by the Fur Locus. J. Bacteriol..

[130] Svinarich D.M., Palchaudhuri S. (1992). Regulation of the SLT-1A toxin operon by a ferric uptake regulatory protein in toxinogenic strains of Shigella dysenteria type 1. J. Diarrhoeal Dis. Res..

[131] Marteyn B., West N.P., Browning D.F., Cole J.A., Shaw J.G., Palm F., Mounier J., Prevost M.-C., Sansonetti P., Tang C.M. (2010). Modulation of *Shigella* Virulence in Response to Available Oxygen In Vivo. Nature.

[132] Vergara-Irigaray M., Fookes M.C., Thomson N.R., Tang C.M. (2014). RNA-Seq Analysis of the Influence of Anaerobiosis and FNR on *Shigella flexneri*. BMC Genom..

[133] Mitobe J., Morita-Ishihara T., Ishihama A., Watanabe H. (2008). Involvement of RNA-Binding Protein Hfq in the Post-Transcriptional Regulation of InvE Gene Expression in *Shigella sonnei*. J. Biol. Chem..

[134] Mitobe J., Yanagihara I., Ohnishi K., Yamamoto S., Ohnishi M., Ishihama A., Watanabe H. (2011). RodZ Regulates the Post-Transcriptional Processing of the *Shigella sonnei* Type III Secretion System. EMBO Rep..

[135] Standish A.J., Teh M.Y., Tran E.N.H., Doyle M.T., Baker P.J., Morona R. (2016). Unprecedented Abundance of Protein Tyrosine Phosphorylation Modulates *Shigella flexneri* Virulence. J. Mol. Biol..

[136] Getz L.J., Runte C.S., Rainey J.K., Thomas N.A. (2019). Tyrosine Phosphorylation as a Widespread Regulatory Mechanism in Prokaryotes. J. Bacteriol..

[137] Weatherspoon-Griffin N., Wing H.J. (2016). Characterization of SlyA in *Shigella flexneri* Identifies a Novel Role in Virulence. Infect. Immun..

[138] Lithgow J.K., Haider F., Roberts I.S., Green J. (2007). Alternate SlyA and H-NS Nucleoprotein Complexes Control HlyE Expression in *Escherichia coli* K-12. Mol. Microbiol..

